# Monitoring Ethylene Oxide Residues in Food: A Simplified QuEChERS-Based GC-MS/MS Method for Routine Analysis

**DOI:** 10.3390/molecules31111978

**Published:** 2026-06-05

**Authors:** Tabita Mauti, Daniela Delfino, Valentina Nicolini, Barbara Droghei, Daniele Colangelo, Daniela Triolone, Fulvia Fiorucci, Paolo Di Giustino, Marta Mancuso, Marianna Leo, Francesca D’Onofrio, Alessandro Ubaldi, Katia Russo

**Affiliations:** 1 Istituto Zooprofilattico Sperimentale del Lazio e della Toscana M. Aleandri, Via Appia Nuova 1411, 00178 Rome, Italy; tabita.mauti@izslt.it (T.M.); daniela.delfino@izslt.it (D.D.); valentina.nicolini-esterno@izslt.it (V.N.); barbara.droghei@izslt.it (B.D.); daniele.colangelo@izslt.it (D.C.); daniela.triolone@izslt.it (D.T.); fulvia.fiorucci@izslt.it (F.F.); paolo.digiustino@izslt.it (P.D.G.); marta.mancuso@izslt.it (M.M.); marianna.leo-esterno@izslt.it (M.L.); francesca.donofrio@izslt.it (F.D.); alessandro.ubaldi@izslt.it (A.U.); 2 School of Specialisation in Food Science, Tor Vergata University of Rome, 00133 Rome, Italy

**Keywords:** ethylene oxide, 2-chloroethanol, QuEChERS method, food safety, pesticide residue analysis, food contaminants, method validation

## Abstract

Ethylene oxide (EtO) has been banned in the European Union since 1991 as a fumigant for food commodities. Nevertheless, recurrent contamination incidents, especially since 2020, involving imports from India, have raised significant food safety concerns. Despite regulatory measures, EtO and its metabolite, 2-chloroethanol (2-CE), continue to be detected in a variety of food products, including dried foods, dietary supplements, and food additives. This study presents a QuEChERS-based method involving the conversion of EtO into 2-CE, which is subsequently quantified by isotope dilution gas chromatography–tandem mass spectrometry (GC-MS/MS). In contrast to previously published methods, this protocol utilises an Agilent HP-5ms Ultra Inert column (30 m × 250 μm × 0.25 mm), routinely employed in our laboratory for multi-residue pesticide analysis. The proposed approach is therefore readily adaptable to laboratories already performing multi-residue analyses, as it does not require modifications to existing instrumental configurations. The method was validated in accordance with SANTE/11312/2021 guidelines. A total of 84 samples, primarily imported from India, as well as from Brazil, Argentina, and the United Kingdom, were analysed. 2-CE was detected in four samples, and in two cases, the sum of EtO and 2-CE, expressed as EtO, exceeded the European Union (EU) maximum residue limit (MRL).

## 1. Introduction

EtO is a colourless, flammable, low-molecular-weight gas, and the simplest epoxide. It is predominantly synthesised as a precursor to various industrial chemicals, including ethylene glycol, glycol ethers, ethanolamines, and ethoxylates [1,2,3]. EtO exhibits excellent penetration properties, making it highly effective at inactivating a broad range of microorganisms, including fungi, bacteria and insects [1,2]. Its strong antimicrobial activity has led to its extensive use as a sterilising agent for medical devices and as a fumigant for various food commodities [4].

Although less than 1% of global EtO production is used for fumigation, its presence in food has raised regulatory and toxicological concerns [5]. EtO is a potent alkylating agent that damages nucleic acids and proteins, resulting in mutagenic, carcinogenic, and reproductive effects [4]. Studies conducted by Agency for Toxic Substances and Disease Registry (ATSDR) and the U.S. Environmental Protection Agency (EPA) have confirmed EtO’s toxicity, particularly via inhalation exposure, affecting the respiratory, haematological, neurological, endocrine, and reproductive systems. Furthermore, EtO is classified as a Group 1 human carcinogen by the International Agency for Research on Cancer (IARC) [4,6].

In food matrices, EtO undergoes rapid transformation reactions and, in the presence of nucleophiles such as water, chloride ions (Cl^−^), and amino groups, it forms more stable derivatives, including ethylene glycol, hydroxyethyl adducts, and 2-CE [5,7]. Among these products, the formation of 2-CE is widely used as an indirect marker of EtO fumigation [4]. Consequently, consumer exposure mainly involves EtO reaction products, since residual EtO in food can be converted into 2-CE under acidic gastric conditions [5]. Experimental studies suggest that this compound has carcinogenic and reproductive toxicity potential [7].

For these reasons, the use of EtO in food products as a food fumigant has been banned across all EU countries since 1991 [5,7]. However, it remains authorised for certain applications in countries such as the United States, Canada, and India, creating significant challenges for international trade [8].

Since 2020, multiple Rapid Alert System for Food and Feed (RASFF) notifications, first issued by Belgian authorities, have reported excessive EtO residues in sesame seeds imported from India, leading to large-scale recalls of sesame-containing products [5,7,8]. In response, the European Commission adopted Regulation (EU) 2020/1540 [9], which requires reinforced border controls, with 50% of sesame consignments from India subject to inspection [7].

According to EU legislation, residues are defined as the sum of EtO and 2-CE, expressed as EtO. The maximum residue limits (MRLs) are set at 0.05 mg/kg for nuts, oilseeds and oil fruits, 0.1 mg/kg for teas, cocoa and spices, and 0.02 mg/kg for other commodities [10]. Ultimately, Commission Regulation (EU) 2022/1396, which lays down specifications for food additives and establishes a maximum limit of 0.1 mg/kg in these matrices [11].

Despite these measures, EtO contamination continues to be reported in various food matrices, including spices (e.g., ginger, caraway, white pepper), herbal teas (e.g., chamomile), dietary supplements, and food additives [12]. The main analytical challenges include high volatility of EtO, matrix effects particularly in dry and oily foods, and potential analyte loss during sample preparation [7]. Moreover, previous studies have reported that EtO rapidly dissipates during ventilation, shipment, and storage, whereas 2-CE can persist as the main residue of toxicological concern in food products [13].

Therefore, this ongoing situation highlights the need for reliable and robust analytical methods capable of detecting EtO and its proxy compound, 2-CE, in diverse and complex food matrices. In this context, several studies have described analytical methods for the determination of EtO using chromatographic columns specifically designed for volatile organic compounds (VOCs). Owing to the high volatility and low boiling point of EtO, these columns are commonly selected to ensure adequate chromatographic separation [4,5,13].

Table 1 summarises studies published over the last five years. In particular, the table reports the main characteristics of the analytical methods, including the type of chromatographic column used, the analytical technique applied, and the food matrices analysed.

Various analytical strategies were developed for the determination of EtO and 2-CE in food matrices. The most widely used approaches were based on the conversion of EtO to 2-CE, followed by extraction using the QuEChERS method and GC-MS/MS analysis. Alternative methodologies included a dynamic headspace-GC-MS/MS method for the simultaneous determination of two analytes. More recently, automated headspace techniques with trap enrichment were introduced [2,7,8,13,14,15,16].

Within the framework of official controls, the aim of this work was to enable the detection of EtO in different food matrices by adapting the method to the requirements of our laboratory, which is routinely involved in multi-residue pesticides analysis, while maintaining established analytical workflows. Thus, a QuEChERS-based method involving the conversion of EtO into its proxy analyte, 2-CE, followed by quantification via isotope dilution GC-MS/MS, was validated. The method employed a GC column commonly used in laboratories involved in multi-residue pesticide analysis, thereby ensuring compatibility with routine analytical workflows. Validation was performed on three representative commodity groups, namely high-water-content matrices, difficult or unique commodities, and high oil/fat-content and very low-water-content matrices. Furthermore, this study reports monitoring data collected over the past two years (January 2024–January 2026) from various food matrices to provide a comprehensive overview of contamination.

## 2. Results and Discussion

The present study aimed to evaluate an alternative analytical approach for determining EtO and 2-CE using an Agilent HP-5ms Ultra Inert column (30 m × 250 μm × 0.25 μm), which is routinely employed in our laboratory for multi-residue analysis. The choice of the column was driven by the need to determine the target analytes without modifying the existing chromatographic setup. Indeed, using a column specifically designed for volatile compounds would have resulted in frequent column replacements, longer analysis times, and substantial changes to laboratory workflows. Therefore, a QuEChERS-based method was employed and combined with isotope-dilution GC–MS/MS.

**Figure 1:**
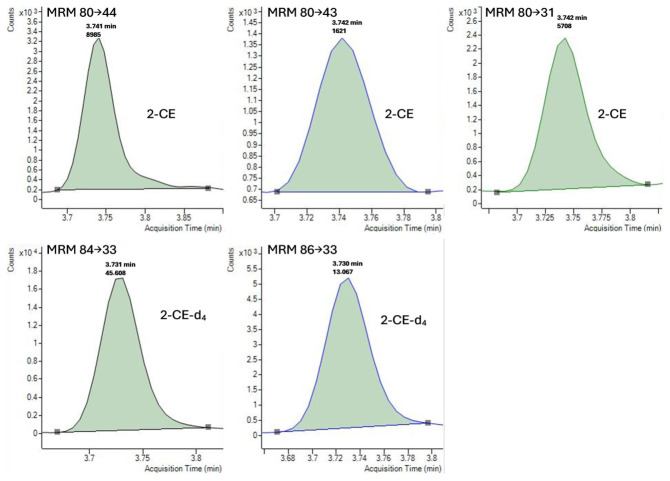
GC–MS/MS chromatographic profiles obtained for 2-CE and the internal standard 2-CE-d_4_ in an Okra sample fortified at the limit of quantification (LOQ), using an HP-5ms Ultra Inert column (30 m × 0.25 mm × 0.25 μm). The upper panels show the chromatographic transitions of 2-CE (*m*/*z* 80 → 44, 80 → 43, and 80 → 31), whereas the lower panels show the transitions of 2-CE-d_4_ (*m*/*z* 84 → 33 and 86 → 33). Different line colours represent different MRM transitions. The dots indicate the start and end points of peak integration used to define the integration boundaries for peak area calculation.

Figure 1 shows the GC-MS/MS chromatogram obtained from an Okra sample spiked at the limit of quantification (LOQ).

Quantification of the analytes was performed in multiple reaction monitoring (MRM) mode using the isotope-dilution method, improving selectivity, specificity, and sensitivity. The MRM transitions were selected based on data reported in recent literature [4,5,7]. The instrumental parameters are reported in Table 2.

### 2.1. Method Validation Performance

The method was validated in accordance with the European guidelines SANTE/11312/2021 [17] and Regulation (EC) No. 396/2005 [18], evaluating the LOQ, linearity range, specificity, selectivity, accuracy and precision under repeatability conditions, including the relative standard deviations (RSD%) and measurement uncertainty. To carry out the validation procedure, three representative matrices were selected: chili peppers (high–water-content matrix), dried bay laurel (difficult matrix) and sesame seeds (high oil/fat content and very low water matrix). For each matrix, at least five replicates were analysed at the LOQ and five replicates at 10 × LOQ.

The analytical approach is based on quantitative conversion of EtO into its proxy compound, 2-CE. To assess the applicability of the method, EtO was spiked into chilli pepper samples at a concentration of 0.010 mg/kg. The concentration of 2-CE measured in the fortified samples was used to estimate the original EtO content. A conversion factor of 0.55 was applied to back-calculate EtO from the detected 2-CE, in agreement with the stoichiometric relationship reported by the European Food Safety Authority (EFSA) [19]. The experiment confirmed complete transformation of EtO into 2-CE under the adopted extraction and derivatization conditions.

The sample pretreatment procedure is described in detail in Section 3.5 and was optimised in terms of extraction efficiency and purification strategy. In particular, experiments were conducted both with and without anhydrous MgSO_4_ to evaluate its effect on phase separation, residual water removal, and analytical recovery. The best recoveries and overall analytical performance were obtained when MgSO_4_ was included in the extraction procedure. Furthermore, different purification compositions were evaluated according to matrix characteristics. In addition, different inlet liner configurations were assessed, and the Agilent 5183-4647 Ultra Inert split liner with glass wool and low-pressure drop was selected because it provided improved chromatographic stability and better reproducibility for the target analytes.

#### 2.1.1. Linearity and Limit of Quantification (LOQ)

Quantification was performed using isotopic dilution with matrix-matched calibration curves consisting of five calibration levels within the investigated ranges (0.008–0.124 mg/kg for chilli peppers, 0.083–1.24 mg/kg for dried bay laurel leaves, and 0.013–0.250 mg/kg for sesame seeds). In all cases, the correlation coefficients were at least 0.995. Prior to extraction, calibration standards were prepared by spiking blank matrix samples with the target analyte and the isotopically labelled internal standard. Blank matrices were previously verified to be free of interfering peaks at the retention times of both the target compounds and the internal standards. Calibration curves were constructed by plotting the analyte-to-internal standard peak area ratio against the corresponding analyte-to-internal standard concentration ratio. In all cases, the correlation coefficients were at least 0.995. Furthermore, the deviation between the back-calculated concentration and the nominal concentration of each calibration standard did not exceed ±20%. Since ethylene oxide is completely converted into 2-CE, the final concentration, expressed as EtO equivalents in the sample, was calculated as follows:

EtO (sum) (mg/kg) = [2-CE] × 0.55

The LOQs were verified and established at 0.010, 0.100, and 0.025 mg/kg for EtO, and 0.018, 0.180 and 0.045 mg/kg for 2-CE, in high-water-content commodities, difficult and/or unique matrices, high oil/fat content, low water content, respectively.

#### 2.1.2. Specificity and Selectivity

Specificity and selectivity were evaluated by analysing ten blank samples fortified with isotopically labelled internal standards (2-CE-d_4_ and EtO-d_4_). No interfering signals were observed at the retention times of the target analytes, confirming the absence of matrix-derived interferences.

#### 2.1.3. Precision and Accuracy

Precision was evaluated using fortified samples of chilli peppers, dried bay laurel leaves, and sesame seeds.

For chilli peppers, the fortification levels were as follows: LOQ level (EtO 0.010 mg/kg and 2-CE 0.018 mg/kg) and Level 1 (EtO 0.100 mg/kg and 2-CE 0.180 mg/kg).

For dried bay laurel leaves, the fortification levels were: LOQ level (0.10 mg/kg EtO and 0.180 mg/kg 2-CE) and Level 1 (1.00 mg/kg EtO and 1.80 mg/kg 2-CE).

For sesame seeds, the fortification levels were: LOQ level (EtO 0.025 mg/kg and 2-CE 0.045 mg/kg) and Level 1 (EtO 0.250 mg/kg and 2-CE 0.450 mg/kg).

For all fortification levels, RSD% complied with the acceptance criteria defined by the SANTE guidelines, with values ≤ 20% [17].

Accuracy was assessed through recovery experiments by comparing the measured concentrations in fortified samples with the corresponding spiked levels. Recoveries for both EtO and 2-CE were within the acceptable ranges specified by the SANTE guidelines (70–120%), ranging from 94% to 118% (EtO) and from 92% to 119% (2-CE) [17].

The results obtained demonstrate excellent analytical performance, confirming that the selected chromatographic column provides reliable determination of EtO (quantified as 2-CE) and 2-CE. All validation parameters met the requirements set by SANTE guidelines. The results are summarized in Table 3.

#### 2.1.4. Ongoing Validation

Ongoing method validation, according to Appendix A of the SANTE document [17], was carried out by analysing spiked samples included in routine analytical batches.

In this context, recovery and within-laboratory reproducibility (RSDwR) were continuously monitored to confirm method robustness and to verify that minor procedural adjustments did not affect analytical performance. To further illustrate the method’s performance, data are reported for eight fortified samples from the ‘difficult/unique commodities’ category. These samples were analysed during eight independent analytical sessions conducted by different operators. The method demonstrated an average recovery rate of 99% and a RSD% of 12.41%, confirming satisfactory robustness and consistency of analytical performance under routine operating conditions.

#### 2.1.5. Measurement Uncertainty

The measurement uncertainty was estimated as required by ISO/IEC 17025:2017 [20] and the SANTE/11312/2021 guidelines [17]. A bottom-up approach was applied, in which individual uncertainty contributions (repeatability, calibration, weighing, volume, and recovery), determined during method validation under intra-laboratory reproducibility conditions, were combined using the root-sum-of-squares method. A coverage factor of k = 2 (95% confidence level) was applied to obtain the expanded uncertainty. The resulting U′ values were below 50% (Table 4), complying with the SANTE performance criteria.

Therefore, the proposed method demonstrated excellent analytical performance, providing sufficient sensitivity to quantify EtO and 2-CE in the tested food matrices.

### 2.2. Real Samples

To evaluate the applicability of the validated method, 84 real samples, primarily originating from India, were collected and analysed within the framework of official control activities at Border Control Posts (BCPs). Table 5 summarises the distribution across different food categories and matrices, showing both the total number of samples analysed and their corresponding percentages relative to the overall sample size.

Overall, the majority of samples were derived from Fruits and Vegetables, particularly okra, which accounted for 39 samples (46%), representing by far the most prevalent matrix. Within the category Dietetic Foods, Food Supplements and Fortified Foods, herbal supplements were the most represented subgroup (12%), and while in the Herbs and spices category, dried chilli showed a relatively high frequency (8.3%). The remaining matrices were present in low proportions (1.2–4.8%).

As reported in Table 6, out of the 84 samples analysed, 2-CE levels were below the LOQ in 80 samples (95%), indicating the absence of quantifiable residues in the majority of the analyzed matrices and general compliance with EU legislation. One chamomile sample showed a detectable signal (>LOQ); however, the amount of sample available did not meet the criteria required for confirmatory analysis. In the remaining three samples (3.6%), 2-CE residues were detected at concentrations ranging from 0.255 to 0.386 mg/kg, suggesting possible prior exposure of the raw materials to EtO treatment.

In two samples-dried bay laurel leaves and a food supplement (2.4%), both originating from India-the residue level reported as the sum of EtO and 2-CE, expressed as EtO, exceeded the maximum residue limit (MRL) established for these compounds (0.050 and 0.1 mg/kg, respectively). These data indicate a generally low occurrence of EtO in the samples analyzed.

In comparison, previous studies have reported various concentration levels of 2-CE and EtO in different products. Lee et al. [13] found high levels of 2-CE in 10 out of 53 spice samples (19%), ranging from 11 to 885 mg/kg, and in 8 out of 106 chilli seasoning samples (7.5%), ranging from 16 to 186 mg/kg, indicating prior EO treatment. Nerpagar et al. [15] reported EtO and 2-CE residues at much lower levels in turmeric, cumin, and sesame powders (0.028–0.034 mg/kg), while locust bean gum and ashwagandha showed no detectable residues. In addition, Bessaire et al. [8] analyzed 23 ice cream samples and detected EtO in 16 samples, with concentrations ranging from 0.021 to 0.052 mg/kg.

Moreover, these findings highlight the need for enhanced detection and monitoring systems to ensure food safety and compliance with regulatory standards, as reflected both in the results of the present study and in those reported in the literature, as well as in RASFF notifications, which frequently report non-compliant levels in spices, herbs and dietary supplements.

**Figure 2:**
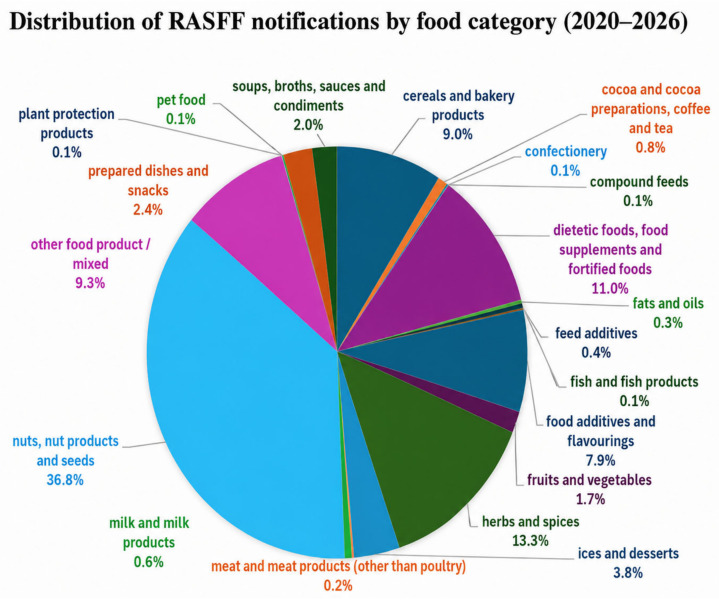
Distribution of RASFF notifications by food category, expressed as a percentage of the total number of notifications for the period from 9 September 2020 to January 2026. (Data retrieved from the RASFF Window accessed on 10 January 2026).

In particular, following the notification issued by the Belgian authorities to the RASFF system in September 2020, the number of EtO-contaminated products reported through the RASFF system increased substantially, reaching more than 1000 notifications to date. Figure 2 shows the distribution of RASFF notifications by food category. The data were extracted from the RASFF Window database on 10 January 2026. The search was conducted using the keyword ‘ethylene oxide’, covering the period from September 2020 to January 2026. The notifications were then manually classified according to the RASFF food categories available in the database and aggregated by year and product category for descriptive analysis. A large proportion of notifications concerned nuts, nut products, and seeds (36.8% of total cases), followed by herbs and spices (13.3%) and dietetic foods, food supplements, and fortified foods (11.0%). Minor contributions were observed for categories such as milk and dairy products, feed additives, cocoa and coffee products, and others, each accounting for less than 1% of total notifications.

**Figure 3:**
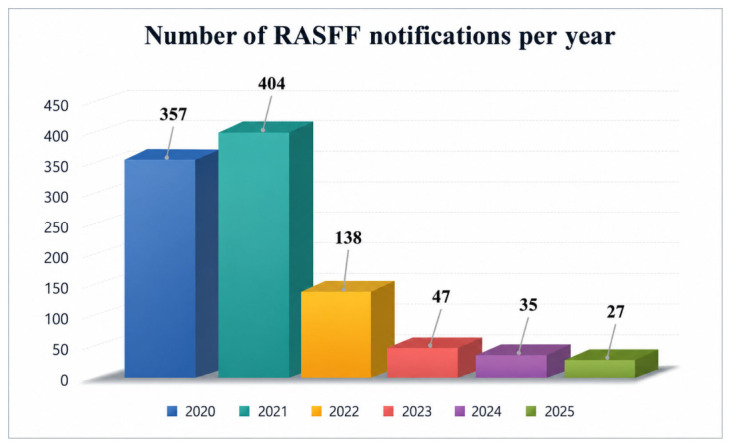
Number of RASFF notifications per year (2020–2025).

A marked increase in notifications was observed in 2021, reflecting increased control activities and monitoring by the competent authorities following the initial detection of EtO contamination. In subsequent years, a gradual decline in notifications was recorded, with a substantial reduction already evident in 2022 and continuing over the following three years (Figure 3). This trend may reflect the impact of regulatory measures and reinforced monitoring activities, together with improved compliance by exporting countries.

## 3. Materials and Methods

### 3.1. Chemicals and Reagents

All solvents used in this study were for pesticide residue trace analysis. Acetonitrile (ACN) and hydrochloric acid (HCl) were purchased from Carlo Erba (Milan, Italy). Anhydrous magnesium sulphate (MgSO_4_) was provided by Carlo Erba (Milan, Italy) and ready-to-use QuEChERS salts were supplied by Agela Technologies (Tianjin, China) and by Phenomenex (Torrence, CA, USA). Labelled standards 2-CE-d_4_ (1000 μg/mL in methanol) and EtO-d_4_ (1000 μg/mL in methylene chloride stabilised in 0.1% hydroquinone) were purchased from CIL (Andover, MA, USA). Native compounds 2-CE (1000 μg/mL in methanol) and EtO (10,000 μg/mL in dimethyl sulphoxide) were obtained from Dr. Ehrenstorfer (Augsburg, Germany).

### 3.2. Apparatus

Analyses were performed using an Agilent 8890 GC system coupled to a 7010C triple quadrupole mass spectrometer and equipped with a 7693A autosampler (Agilent Technologies, Santa Clara, CA, USA). Chromatographic separation was achieved using an Agilent HP-5ms Ultra Inert column (30 m × 0.25 mm × 0.25 μm; Agilent Technologies, Santa Clara, CA, USA). Injection was carried out using a multimode inlet (MMI) operating in split mode (split ratio 4:1). The MMI temperature program was as follows: initial temperature 90 °C (0.8 min); ramped at 720 °C/min to 250 °C, hold time 10 min. Injection volume was 2 μL. Agilent 5183-4647 Ultra Inert split liner with glass wool and low-pressure drop was used (Agilent Technologies, Santa Clara, CA, USA).

The oven temperature program was set as follows: 40 °C (held for 2.5 min), ramped at 25 °C/min to 130 °C, then at 50 °C/min to 280 °C (held for 8 min), for a total run time of 17.1 min. Helium (He) was used as the carrier gas at a linear flow rate of 0.98 mL/min.

The mass spectrometer operated in electron ionisation (EI) mode at 70 eV, with source and transfer line temperatures of 280 °C. Nitrogen (1 mL/min) was used as the collision gas and He (4 mL/min) as the quench gas. Data were acquired in Multiple Reaction Monitoring (MRM) mode.

### 3.3. Standard Solution Preparation

Working standard solutions were prepared at low temperature conditions to minimise evaporation losses of EtO and to reduce volumetric errors associated with temperature-dependent density variations in solvents such as ACN [5].

Solutions were obtained by appropriate dilution with ACN, previously placed in the freezer for at least 20 min, to reach final concentrations of EtO (10 µg/mL and 1 µg/mL), 2-CE (1 µg/mL), EtO-d_4_ (10 µg/mL), and 2-CE-d_4_ (10 µg/mL). All working solutions were stored at −20 °C prior to use.

### 3.4. Sample Collection

This study presents data on food samples collected under official control programmes carried out between 2024 and 2026. The samples were analysed by the Istituto Zooprofilattico Sperimentale del Lazio e della Toscana M. Aleandri (IZSLT) and include inspections conducted at Border Control Posts (BCPs), specifically at Fiumicino Airport and Livorno Port, and at Veterinary Offices for the Fulfilment of Community Obligations (UVACs). Method validation was performed using three representative matrices selected in accordance with the requirements of the competent authority and reflecting the main sample typologies collected during official controls.

### 3.5. Sample Preparation

The following sample pretreatment procedure, based on the method reported by Wenio et al. [4] with minor modifications, was employed. Approximately 4 g of homogenised sample (or 2 g for difficult matrices and high oil/fat content and very low water matrix) was weighed into pre-frozen 15 mL polypropylene centrifuge tubes. EtO-d_4_ was added to the sample (44 or 22 µL), and the mixture was briefly vortexed. Ten mL of pre-cooled 0.2 M HCl solution in ACN (*v*/*v*) was added. The mixture was incubated for 30 min at −20 °C in an ice bath to promote the conversion of EtO to 2-CE.

Subsequently, 0.5 g of anhydrous MgSO_4_ was added, and the mixture was shaken before ultracentrifugation at 10,000 rpm for 5 min at 4 °C. After centrifugation, 1 mL of the organic phase was carefully transferred into a clean tube containing ready-to-use QuEChERS salts. In the case of high-water content commodities and high oil/fat content and very low water matrix, a cleanup containing 25 mg PSA, 150 mg MgSO_4_, and 150 mg C18 was used, whereas for difficult matrices a cleanup consisting of 750 mg MgSO_4_, 125 mg PSA, and 12.5 mg GCB was employed. Finally, the resulting solution was collected for GC-MS/MS analysis.

## 4. Conclusions

The determination of EtO and its metabolite, 2-CE, has received significant attention due to public health concerns; therefore, reliable analytical methods are critical.

The analytical approach presented here, based on QuEChERS extraction and combined with isotope-dilution GC-MS/MS, was successfully adapted to the routine requirements of our laboratory for regulatory monitoring of EtO residues across various food matrices. By efficiently integrating into the existing laboratory workflow without requiring modifications to the instrumental setup, the proposed method offers a significant practical advantage. This operational efficiency highlights its potential value for other laboratories involved in official control activities that are required to perform multiresidue analyses.

As part of official control activities, 84 samples were analysed. In line with the decreasing trend observed in recent years in RASFF notifications, most analysed samples showed concentrations below the LOQ. Nevertheless, the presence of the target analytes in four samples from product categories most frequently involved in RASFF notifications confirms the crucial importance of continuous surveillance.

## Figures and Tables

**Table 1:** Overview of recent studies on the determination of EtO and 2-CE residues in food, including the GC columns used, the analytical and extraction methods applied, and the food matrices investigated.

Reference	Column	Analytical Method	Matrices
Lee et al., 2025 [13]	SupelcoWax (10, 30 m × 0.25 mm × 0.25 μm)	QuEChERS extraction and GC-MS/MS	spices, herbs, processed foods
Hearn et al., 2024 [14]	VF-624 GC column (60 m × 0.25 mm × 1.4 μm)	MSE-HS-trap extraction technique	sesame seeds, guar gum
Bessaire et al., 2023 [2]	DB-624 UI (30 m × 0.25 mm × 1.4 µm) or HP-VOC (30 m × 0.20 mm × 1.12 mm)	QuEChERS extraction and GC-MS/MS	herbs, spices, fruits and vegetables, stabilisers
Nerpagar et al., 2023 [15]	Rtx-vmS Gc column (60 m × 0.45 mm, 2.55 μm)	dynamic headspace-GC-MS/MS	cumin, ashwagandha, chilli powder, turmeric powder, guar gum, locust bean gum, and ginger powder
Patil et al., 2023 [7]	HP-Wax column (30 m × 0.25 mm × 0.25 μm)	QuEChERS extraction and GC-MS/MS	sesame, cumin, wheat, tea, spices, herbs, dehydrated fruits, food additives, grapes, tomatoes
Madsen et al., 2022 [16]	CP-Sil 8CB (30 m × 0.32 mm × 1 mm)	SPME-GC-MS	low viscous hydroxypropyl methylcellulose
Bessaire et al., 2021 [8]	DB-624 UI; 30 m × 0.25 mm × 1.4 µm	QuEChERS extraction and GC-MS/MS	ice cream

**Table 2:** MRM transition and MS/MS parameters for EtO and 2-CE and their labeled compounds (d_4_).

Compound Name	Precursor Ion (*m*/*z*)	Product Ion (*m*/*z*)	Dwell (ms)	Collision Energy (eV)
EtO	44	29	79.2	5
EtO	44	28	79.2	5
EtO	44	14	79.2	20
EtO-d_4_	48	30	79.2	5
EtO-d_4_	48	16	79.2	20
2-CE	82	44	65.8	5
2-CE	82	31	65.8	5
2-CE	80	44	65.8	5
2-CE	80	43	65.8	5
2-CE	80	31	65.8	5
2-CE-d_4_	86	33	65.8	5
2-CE-d_4_	84	33	65.8	5

**Table 3:** Validation parameters for EtO (detected as 2-CE) and 2-CE in high water content, difficult/unique and high oil/fat content and very low water content commodities.

Commodity Groups	High Water Content			Difficult or Unique Commodities			High Oil/Fat Content and Very Low Water Content		
	Levels mg/kg	Mean Recovery (%)	RSD%	Levels mg/kg	Mean Recovery (%)	RSD%	Levels mg/kg	Mean Recovery (%)	RSD%
EtO (detected as 2-CE)	0.010 (LOQ)	118	2.87	0.100 (LOQ)	111	3.62	0.025 (LOQ)	97	8.20
	0.100	106	6.06	1.00	110	8.01	0.250	94	2.90
2-CE	0.018 (LOQ)	107	2.27	0.180 (LOQ)	119	5.97	0.045 (LOQ)	100	9.09
	0.180	101	2.70	1.80	111	7.99	0.450	92	3.60

**Table 4:** Measurement uncertainty values obtained for EtO in the analysed matrices at the spiking levels examined. The expanded measurement uncertainty (U, k = 2) was compared with the default value of 50% established by the SANTE guidelines.

Commodities	Compound	Spiking Levels (mg/Kg)	Measurement Uncertainty ^a^	Measurement Uncertainty ^b^
High water content	EtO	0.010	13%	50%
		0.100	15%	
	2-CE	0.018	12%	50%
		0.180	12%	
Difficult or unique commodities	EtO	0.100	46%	50%
		1.00	17%	
	2-CE	0.180	15%	50%
		1.80	17%	
High oil/fat content and very low water content	EtO	0.025	36%	50%
		0.250	13%	
	2-CE	0.045	24%	50%
		0.450	12%	

^a^ Metrological Approach. ^b^ Maximum Standard Uncertainty according to SANTE/11312/2021 [17].

**Table 5:** Distribution of samples analyzed by product category.

Food Category	Matrix	Count (N)	Percentage (%)
Dietetic Foods, Food Supplements and fortified foods	Dietary Supplements	1	1.2%
	Herbal Supplements	10	12%
	Foods for special medical purposes	3	3.6%
	Vitamin-Mineral Supplements	1	1.2%
Fruits and Vegetables	Drumstick	1	1.2%
	Okra	39	46%
	Papaya	1	1.2%
Herbs and Spices	Black Pepper	2	2.4%
	Chamomile	1	1.2%
	Dried Bay Laurel	1	1.2%
	Dried Chilli	7	8.3%
	Fenugreek Seeds	1	1.2%
	Turmeric powder	4	4.8%
	Turmeric (Root)	1	1.2%
Nuts, nut products and seeds	Cumin Seeds	3	3.6%
	Mustard Seeds	1	1.2%
	Sesame Seed	4	4.8%
Other food products/ mixed	Bulk Liquid Herbal Extracts	1	1.2%
	Pharmaceutical products	2	2.4%

**Table 6:** Results of EtO residues (sum of EtO and 2-CE expressed as EtO, mg/kg) in various commodity matrices from different countries, obtained from official control samples collected between 2024 and January 2026.

Matrix	Country of Origin	Positive Samples	Sum of EtO and 2-CE, Expressed as EtO (mg/kg)
Black Pepper	India	0	<LOQ
Bulk Liquid Herbal Extracts	India	0	<LOQ
Chamomile	United Kingdom	1 (Suspected)	>0.1 *
Cumin Seeds	India	0	<LOQ
Dietary Supplements	India	0	<LOQ
Dried Bay Laurel	India	1	**0.160**
Dried Chilli	India	0	<LOQ
Drumstick	India	0	<LOQ
Fenugreek Seeds	India	0	<LOQ
Foods for Special Medical Purposes	India	1	**0.140**
Herbal Supplements	India	1	**0.212**
Mustard Seeds	India	0	<LOQ
Okra	India	0	<LOQ
Papaya	Brazil	0	<LOQ
Pharmaceutical Products	India	0	<LOQ
Sesame Seeds	India/Argentina	0	<LOQ
Turmeric (Root)	India	0	<LOQ
Turmeric Powder	India	0	<LOQ
Vitamin–Mineral Supplements	India	0	<LOQ

* Detected, but the sample amount was insufficient for confirmatory analysis.

## Data Availability

The original contributions presented in this study are included in the article. Further inquiries can be directed at the corresponding author.
